# *In-vivo* waveguide cardiac magnetic resonance elastography

**DOI:** 10.1186/1532-429X-17-S1-P35

**Published:** 2015-02-03

**Authors:** Ria Mazumder, Bradley D Clymer, Richard D White, Anthony Romano, Arunark Kolipaka

**Affiliations:** 1 Electrical and Computer Engineering, The Ohio State University, Columbus, OH, USA; 2 Naval Research Laboratory, Washington, DC, USA; 3 Department of Internal Medicine-Division of Cardiology, The Ohio State University, Columbus, OH, USA; 4 Department of Radiology, The Ohio State University, Columbus, OH, USA

## Background

Myocardial stiffness (MS) is elevated in heart failure with preserved ejection fraction(HFPEF)[1]. In addition, stiffness elevation in HFPEF exhibits directional dependency[2]. Conventional determinants of MS such as pressure-volume relationship and mechanical testing are invasive and hence clinically inefficient. Therefore, there is a need to non-invasively estimate anisotropic MS to assist in diagnosis and prognosis of HFPEF. In this study we implement waveguide cardiac magnetic resonance elastography (CMRE)[3] to demonstrate the feasibility of estimating anisotropic MS non-invasively in an *in-vivo* porcine model.

## Methods

Waveguide CMRE involves performing diffusion tensor imaging (DTI) in conjunction with conventional CMRE. *In-vivo* CMRE was performed on a pig in a 1.5T MRI scanner. CMRE imaging parameters: TE/TR=9.7/21.4; flip angle 25^○^; mechanical frequency 80 Hz; encoding frequency 160 Hz. Post CMRE acquisition the heart was arrested in diastole using potassium chloride and in-situ cardiac DTI was performed. Cardiac DTI parameters: TE/TR=80/3200; flip angle 90^○^; b-value=0/1000 s/mm^2^; number of directions=12; number of averages=10. CMRE and DTI was performed at the same resolution and the parameters were FOV=320mm^3^; imaging matrix 128x128; slice thickness=2.5mm; DTI was registered with CMRE to exactly match the voxel information from both sets of acquisition. Then both CMRE and DTI were masked to extract the left ventricle. Masked images were processed to estimate i) principle eigenvectors from DTI data sets; ii) and first harmonic displacements from CMRE wave data. Next, a spatial spectral filter was applied on the first harmonic displacement data to isolate waves traveling in particular directions defined by the principle eigenvector. Simultaneously, Helmholtz decomposition was performed to separate the filtered displacements into its longitudinal and transverse components. An orthotropic inversion [3] was performed to calculate compressional (C_11_,C_22_,C_33_) and shear (C_44_,C_55_,C_66_) stiffness coefficients.

## Results

**Figure 1:**
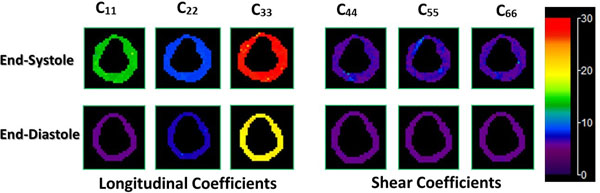
Anisotropic stiffness maps for end-systole (top row) and end-diastole (bottom row).

Figure 1 shows stiffness maps for end-systole and end-diastole. The mean and SD of the compressional and shear stiffness coefficients is listed in Table 1. We have observed that compressional stiffness is higher than shear stiffness. In addition both compressional and shear stiffness coefficients are higher in end-systole as compared to end-diastole.

**Table 1:** Anisotropic Stiffness Measurements

	Compressional Stiffness Coefficients			Shear Stiffness Coefficients		
	C11	C22	C33	C44	C55	C66

End-Systole	13.97 ± 0.38	8.68 ± 0.19	27.34 ± 1.02	6.57 ± 1.23	6.44 ± 0.62	6.45 ± 0.68

End-Diastole	5.49 ± 0.07	7.02 ± 0.22	20.00 ± 0.54	5.52 ± 0.31	5.56 ± 0.39	5.53 ± 0.31

## Conclusions

We have demonstrated the feasibility of estimating *in-vivo* anisotropic stiffness using CMRE. However further validation and application in a diseased model is required.

## Funding

This study has been supported by AHA 13SDG14690027.
